# How can we recognize continuous quality improvement?

**DOI:** 10.1093/intqhc/mzt085

**Published:** 2013-12-04

**Authors:** Lisa Rubenstein, Dmitry Khodyakov, Susanne Hempel, Margie Danz, Susanne Salem-Schatz, Robbie Foy, Sean O'Neill, Siddhartha Dalal, Paul Shekelle

**Affiliations:** 1 The RAND Corporation, 1776 Main Street, PO Box 2138, Santa Monica, CA 90401, USA; 2 Veterans Affairs Greater Los Angeles at Sepulveda, 16111 Plummer Street (152), North Hills, CA 91343, USA; 3 Department of Medicine and School of Public Health, University of California, Los Angeles, CA 90024, USA; 4 Independent Consultant, HealthCare Quality Initiatives, Newton, MA 02459, USA; 5 Leeds Institute of Health Sciences, University of Leeds, Leeds LS2 9JT, UK; 6 Feinberg School of Medicine, Northwestern University, Arthur J. Rubloff Building 420 East Superior Street, Chicago, IL 60611, USA; 7 Veterans Affairs Greater Los Angeles Healthcare System, 11301 Wilshire Boulevard, Los Angeles, CA 90073, USA

**Keywords:** continuous quality improvement, quality improvement, consultants, health care organization

## Abstract

**Objective:**

Continuous quality improvement (CQI) methods are foundational approaches to improving healthcare delivery. Publications using the term CQI, however, are methodologically heterogeneous, and labels other than CQI are used to signify relevant approaches. Standards for identifying the use of CQI based on its key methodological features could enable more effective learning across quality improvement (QI) efforts. The objective was to identify essential methodological features for recognizing CQI.

**Design:**

Previous work with a 12-member international expert panel identified reliably abstracted CQI methodological features. We tested which features met rigorous a priori standards as essential features of CQI using a three-phase online modified-Delphi process.

**Setting:**

Primarily United States and Canada.

**Participants:**

119 QI experts randomly assigned into four on-line panels.

**Intervention(s):**

Participants rated CQI features and discussed their answers using online, anonymous and asynchronous discussion boards. We analyzed ratings quantitatively and discussion threads qualitatively.

**Main outcome measure(s):**

Panel consensus on definitional CQI features.

**Results:**

Seventy-nine (66%) panelists completed the process. Thirty-three completers self-identified as QI researchers, 18 as QI practitioners and 28 as both equally. The features ‘systematic data guided activities,’ ‘designing with local conditions in mind’ and ‘iterative development and testing’ met a priori standards as essential CQI features. Qualitative analyses showed cross-cutting themes focused on differences between QI and CQI.

**Conclusions:**

We found consensus among a broad group of CQI researchers and practitioners on three features as essential for identifying QI work more specifically as ‘CQI.’ All three features are needed as a minimum standard for recognizing CQI methods.

## Introduction

Continuous quality improvement (CQI) methods revolutionized perspectives on healthcare quality in the early 1990s and have since been considered a healthcare industry standard [1–3]. Internal medicine core competencies, for example, indicate that ‘the sponsoring institution and participating sites must demonstrate that there is a culture of patient safety and continuous quality improvement …’[4]. CQI approaches are also often referenced in work focused on developing countries, where scarce medical resources may make innovative adaptation to local circumstances particularly critical [5]. As reflected in the published literature, however, the scientific use and reporting of CQI methods are heterogeneous. Lack of consistency in identifying whether and when improvement initiatives have used CQI has meant that relevant evidence reviews are difficult to interpret, results of individual initiatives that reference use of CQI are difficult to apply, and CQI authors and educators struggle with how to report and reference their methods.

The term CQI does not refer to all QI methods, but rather to those derived from the total quality management (TQM) approach developed by Deming, Juran and others for industrial settings [6]. Healthcare experts such as Berwick, Kaluzny and McLaughlin [7–9] re-envisioned TQM as CQI, reflecting adaptations for better application within healthcare organizations. These methods span a wide variety of possible CQI elements including, for example, plan-do-study-act (PDSA) cycles or diagnostic methods, such as those used for root cause analyses of adverse clinical events [10]. The work presented here aims to anchor use of the term CQI to a clear and parsimonious set of essential features. Additional CQI-related features can then vary to reflect an initiative's purpose, while still enabling recognition of CQI methods.

General CQI and TQM principles and philosophies have infused a wide variety of specific approaches to facilitate their practical application, including, for example, Lean [11], Six Sigma [12], Institute for Healthcare Improvement's Breakthrough Series [13, 14] and 10 0000 Lives and 5 Million Lives campaigns [15, 16] and System Redesign [17]. While the availability of such approaches likely strengthens the field by offering practical alternatives that may fit particular needs and preferences, this broad evolution has created a confusing blend of terminologies.

Moreover, current published definitions of CQI are not adequate for distinguishing CQI efforts from other QI and patient safety intervention approaches [18, 19]. Short definitions aimed at orienting readers to the field in general are broad and vague [20]. Long definitions aimed at helping people carry out CQI successfully cover pages of text and include a wide variety of elements from philosophies to tools, to data sources [9]; no one initiative is likely to use all of these. Therefore, as is the case for other complex interventions [21], defining key theoretical and operational features is critical for building a cumulative science of improvement [22–24]. The set of such features should be broad enough that QI efforts not meeting the definition can be excluded as not CQI, narrow enough that efforts using the features can be included as applications of CQI and specific enough for reliable abstraction from the literature.

In this study, recognized QI experts identified essential *features that define CQI as a method*, cutting across specific named approaches. Some potential CQI features may be attractive philosophically, but less useful for recognizing work as CQI. Previously, for example, we found that ‘continuous’ improvement was not endorsed as essential by in-person panelists, who thought that a time-limited effort should potentially qualify as CQI [19, 25].

To identify the parsimonious set of essential CQI elements reported in this study, we used rigorous, innovative online expert panel [25] and literature review methods. We further contextualized our results based on qualitative analysis of online discussion. Our findings reflect broad consensus among experts on essential CQI features that can be abstracted reliably from the literature when reported by authors. We consider features that cannot be reliably abstracted when reported to be either too complex or too fluid to be useful for recognizing CQI. Panel results can enable more rapid learning from and across CQI initiatives through improved methodological consistency, reporting and comparative analyses.

## Methods

### Overview and prior studies

This study, which was determined to be exempt from the IRB review by RAND and the Veterans Affairs (VA), builds on prior work carried out as the Evidence Review for Quality Improvement Project, funded by the Robert Wood Johnson Foundation (RWJF), the Agency for Healthcare Quality and Research (AHRQ), the VA and RAND over a series of projects on identifying and assessing QI intervention literature [18, 19, 25–27].

### Study design

Assessment of panel consensus on the essential definitional features of CQI based on a priori consensus standards and an online modified-Delphi process [25].

### Rating process and participants

#### Development of potential features for consensus ratings

We previously engaged a panel of 12 national and international QI experts in rating and refining an initial list of 48 potentially definitional CQI features [19, 27]. Over a 1 year period, the experts completed two online surveys, three telephone discussions and a final in-person meeting during which the panel reached consensus on six top-rated CQI features. Two study team raters tested the reliability of the six items when applied to 106 representative, methodologically diverse QI intervention articles. The article set resulted from an electronic search for QI interventions of any kind [18], random sampling of searched articles and hand screening focused on QI rather than specifically on CQI [27]. Two reviewers then abstracted the 106 articles for the six top-rated CQI features. Percent agreement ranged from 55.7 to 75.5% for the six items, and reviewer-adjusted intra-class correlation ranged from 0.43 to 0.62. Although two-thirds of articles documented at least two features, only 14% included some evidence of all six features. The study team concluded that the sensitivity of a definition based on six features was too low; there was no unbiased way to eliminate features; and additional consensus work was required for a useable definition [19]. Therefore, we used the six features from the prior in-person panel meeting, added five that had been rated highly but had not reached the final top six and conducted the online consensus process using 11 CQI features reported here.

#### Panelist identification

LR and SSS used their professional networks to invite Institute for Healthcare Improvement faculty, members of the editorial boards from leading QI research journals, evaluators of RWJF QI programs, and RAND patient safety and QI experts to participate in this study. Experts were asked to nominate other QI professionals and health services researchers. Out of 259 professionals contacted, 119 (46%) agreed to participate [25].

#### Panelist assignment

We used stratified random sampling to assign participants to one of four panels (A, B, C or D) to make it easier for participants to engage in online discussion and test replicability of panel findings. Panels ranged in size from 19 to 40 experts by design; previous analyses showed that panel size did not have a significant effect on discussion or consensus [25].

#### Panel process

Panelists participated over three 1-week phases using ExpertLens, a RAND-developed modified-Delphi online expert panel system. In Phase I, participants rated the 11 potential CQI features. In Phase II, participants saw their own Phase I responses in relation to their panel's median and quartile results. They then participated in asynchronous, anonymous, un-moderated online discussions of the features and ratings. In Phase III, participants re-answered Phase I questions [25].

### Measures and analysis

#### Panelist characteristics

Participants self-identified on an enrollment form as primarily practitioners, primarily researchers or both equally.

#### Panel rating criteria

To determine whether a feature was *definitional*, panelists answered the following question: ‘How important is this feature to the definition of a CQI initiative?’ In addition, we asked participants ‘How important is the implementation of this feature to the success of a CQI initiative?’ and ‘To maximize the usefulness of a CQI publication, how important is it to report on this feature?’ to determine whether a feature was *important to success* and *important to report*, respectively. Questions had Likert scale response sets, ranging from 1 (not important) to 5 (very important). Participants could suggest new features in Phase I, which were added to the Phase III rating form with the single rating question: ‘Given [the feature], would you exclude a study without this feature from a literature review of CQI initiatives?’

#### Consensus

Our a priori definition [28, 29] of consensus on each individual criterion for each feature based on Phase III results was that at least two-thirds of the members of each of the four panels rated the feature as highly important (i.e. >3 on the 5-point importance scale). If all four panels agreed that a feature was important to definition of CQI, we considered this feature to be an *essential feature of CQI methods*. We also assessed whether features were considered important to success and reporting. As additional context, we report the percent of all panelists, regardless their panel assignment, rating the feature as highly important on these criteria.

#### Qualitative data analysis of discussions

The ExpertLens system recorded the discussion text. DK used MAXQDA, qualitative data analysis software, to analyze the text thematically. Using the hybrid approach to thematic qualitative data analysis [30, 31], DK first coded discussion text deductively by reviewing all comments linked to each of the 11 candidate CQI features. He then coded inductively to identify emerging overarching themes across features. DK, LR and SH reviewed coded data to ensure coding consistency; disagreements were discussed until consensus was achieved.

### Role of the funding source

The sponsors had no role in the design and conduct of the study; collection, management, analysis and interpretation of the data; and preparation, review or approval of this manuscript.

## Results

**Figure 1:**
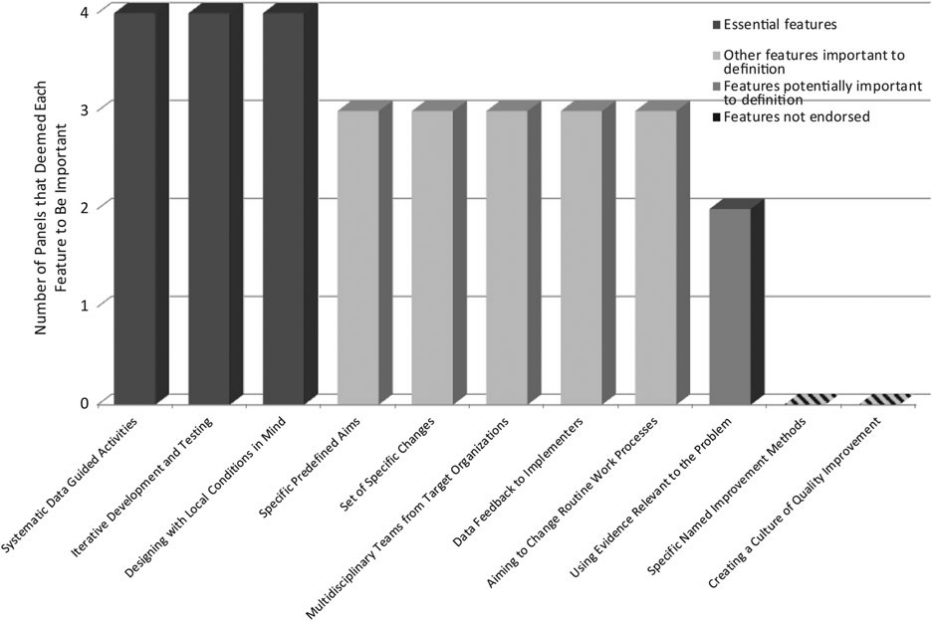
Panel consensus on features important to the definition of CQI. Note: The number of independent panels, where >66.6% of participants deemed a particular feature important for the definition of CQI as a method.

Table 1 shows the list and definitions of potential CQI features rated by the online panels. The level of agreement across panelists and across the four independent panels is shown in Table 2. Three features met our a priori consensus standards, because each of the four panels independently, and all participants taken together, agreed on their importance for defining CQI using the a priori defined cut-off of 66.6%. These *three essential features of CQI methods* are ‘systematic data guided activities,’ ‘designing with local conditions in mind’ and ‘iterative development and testing’ (Fig. 1).

**Table 1:** CQI features used in the study

Feature	Description
1. Systematic Data Guided Activities	Uses systematic data-guided activities (e.g. aims and measures) to achieve improvement
2. Aiming to Change Routine Work Processes	Aims to change how routine or daily care work processes are organized, structured or designed
3. Creating a Culture of Quality Improvement	Seeks to create a culture or mindset of quality improvement
4. Specific Predefined Aims	Seeks to achieve specific pre-identified aims, targets or outcomes
5. Using Evidence Relevant to the Problem	Uses available previously established evidence relevant to the target QI problem or goal (e.g. evidence-based care models or behavioral change strategies)
6. Designing with Local Conditions in Mind	Is designed/implemented with local conditions in mind (i.e. to fit the special characteristics of targeted local environment(s))
7. Iterative Development and Testing	Involves an iterative (more than one cycle) development and testing process such as PDSA
8. Multidisciplinary Teams from Target Organizations	Designed and/or carried out by multidisciplinary teams that include members from the target organizations/communities
9. Data Feedback to Implementers	Involves feedback of data (e.g. quantifiable performance measures/benchmarks) to initiative designers and/or implementers
10. Specific Named Improvement Methods	Identifies the methods used for producing change as CQI or a named, related method (e.g. DMAIC, Define-Measure-Analyze-Improve-Control; ‘six-sigma;’ ‘Toyota production system’) aimed at producing improvement
11. Set of Specific Changes	The initiative seeks to implement a set of specific changes in order to embed improvements in routine or daily care work processes

**Table 2:** Number of panelists rating each feature as highly important on the criterion ‘importance to the definition of a CQI initiative’

Feature	Panelists in Panel A (*N*, %)	Panelists in Panel B (*N*, %)	Panelists in Panel C (*N*, %)	Panelists in Panel D (*N*, %)	Total (*N*, %)
**1. Systematic data guided activities**	**10**	**10**	**32**	**26**	**78**
	**100%**	**100%**	**97%**	**100%**	**99%**
2. Aiming to change routine work processes	9	5	28	18	60
	90%	50%	85%	69%	76%
3. Creating a culture of quality improvement	5	1	19	16	41
	50%	10%	59%	59%	52%
4. Specific predefined aims	8	6	28	24	66
	80%	60%	85%	89%	83%
5. Using evidence relevant to the problem	5	7	23	15	50
	50%	70%	72%	56%	63%
**6. Designing with local conditions in mind**	**9**	**9**	**29**	**24**	**71**
	**90%**	**90%**	**91%**	**89%**	**90%**
**7. Iterative development and testing**	**8**	**9**	**25**	**26**	**68**
	**80%**	**90%**	**76%**	**96%**	**85%**
8. Multidisciplinary teams from target organizations	6	7	21	18	52
	67%	70%	64%	67%	66%
9. Data feedback to implementers	10	10	18	26	64
	100%	100%	62%	96%	84%
10. Specific named improvement methods	4	3	9	13	29
	40%	30%	28%	48%	37%
11. Set of specific changes	10	6	26	19	61
	100%	60%	81%	70%	77%

All four panels independently and all participants taken together consider the bold features important to the definition.

Using the same a priori criteria for panel agreement, panelists considered the three essential CQI features to also be important for CQI success and reporting; data are shown in the online material (Supplementary material Appendix Table 1). Panelists rated only one feature—using ‘specific named improvement methods’—as not important based on any of our rating criteria. Online discussion comments indicated that the terms do not reliably link to what was planned or done, are often renamed for specific healthcare organizations to improve buy-in and are not mutually exclusive.

Among the seven additional features that panel ratings did not identify as definitional, four panels agreed that ‘using evidence relevant to the problem’ is a feature *important to reporting*, whereas ‘specific predefined aims’ and ‘multidisciplinary teams from target organizations’ are the features *important for CQI success* (Supplementary material Appendix 1). All panels agreed that ‘aiming to change routine work’ and providing ‘data feedback to implementers’ are *important to both CQI success and reporting*. Finally, two new features suggested by panelists in Phase I (‘provides data on what was done to produce change’ and ‘describes the context within which the initiative took place’) reached our consensus level of 66.6% across all four panels in Phase III as issues that should be addressed in any CQI publication.

Analysis of the online discussion data summarized in Table 3 shows that although panelists considered all rated features to be relevant to CQI, they struggled with assessing the extent to which all CQI projects must manifest the feature. Participants noted that the importance of some features depends on the scope and complexity of an intervention. For example, if an intervention is limited in scope, successful QI may not require multidisciplinary teams (a feature not achieving consensus as definitional, though still considered important for project success in quantitative analyses). For the features ‘systematic data guided activities,’ ‘aiming to change routine work,’ ‘specific, pre-defined aims’ and ‘having a set of specific changes,’ participants debated possible changes in wording.

**Table 3:** Qualitative analysis of discussion data for each feature

Feature	Panels discussing this feature (no. of comments)	Summary of main themes and comments
1. Systematic data guided activities	A and C (10)	• Should this criterion be distinguishing CQI from research? • Is the use of aims and measures an example of a data-guided activity? *Illustrative Comment*: ‘Decisions should be data-guided, but aims and measures should be established in a manner that facilitates identification, selection, collection and use of relevant data.’
2. Aiming to change routine work	B, C, and D (8)	• Is CQI changing routine practices or introducing new processes? Is there a difference between the two? *Illustrative Comment*: ‘I thought that this might exclude QI efforts that aim to change processes that are not routine/daily (e.g. increasing seasonal flu shots…time limited project) but this would hinge on how you interpret ‘routine/daily’.
3. Creating a culture of quality improvement	A, B, and D (16)	• While culture is often critical to the success and sustainability of CQI, it may not be important to its definition. • Culture change may not be the primary purpose of a QI intervention, although it may be necessary for success. *Illustrative Comment*: ‘Some CQI projects can be initiated and completed without culture change. However, developing an organization that incorporates CQI in all of its work requires culture change.’
4. Specific, pre-defined aims	B and D (11)	• Does having a pre-defined aim imply that issues identified during the implementation will be ignored? • Does ‘pre-defined’ indicate predetermined by others (versus those making the improvements)? • Does this feature lead to incremental versus fundamental or visionary change? *Illustrative Comment:* ‘If you don't have an identified aim, how can you plan the improvement or know if it was successful? ….However, if ‘pre-identified’ means identified by somewhat [stet] in a QI office or at the top of the hierarchy in the organization…I completely agree that this is not an important part of CQI.’
5. Using evidence relevant to the problem	A and C (10)	• While CQI projects that are not based on strong evidence may have less chance of success, improvement may need to happen in the absence of strong evidence. • CQI can be used to generate evidence that an intervention is effective. • In the absence of existing evidence, expert judgment may be used to evaluate the potential of a new initiative. *Illustrative Comment:* ‘The best evidence for the change is whether it is effective in the current context. Prior evidence, if available, should be consulted, but (a) it is not always available and (b) even if available is not always relevant.’
6. Designing with local conditions in mind	B and D (7)	• What are the implications when an intervention is developed elsewhere and implemented locally using CQI? • Do senior management roll-outs of changes across clinics qualify as CQI? *Illustrative Comment:* ‘In collaborative QI projects involving organization from multiple organizations [stet] the design and description of changes is generic and doesn't address local conditions. Then each participating organization tests and adapts the changes to their local conditions.’
7. Iterative development and testing	A, B, and D (11)	• Should implementation of improvements without iterative testing be considered CQI? • If the improvement requires only one cycle of testing, can it be considered CQI? • If an improvement is spread and embedded in practice without iterative testing, can it be considered CQI? *Illustrative Comment:* ‘QI is not equivalent to CQI. It seems to me that one defining feature of CQI to distinguish it from other types of QI activities is the iterative cycle….Implementing someone else's intervention may be important, may be laudable, and may represent a significant challenge, but if it is not done iteratively in some manner of PDCA [Plan-Do-Check-Act] then is it CQI? My vote is no.’
8. Multidisciplinary teams from target organizations	C and D (20)	• Can CQI sometimes be carried out by just one discipline? • Does the scale and scope of the project determine the need for multiple disciplines? • Is involvement of stakeholders equivalent to multidisciplinary teams? *Illustrative Comment:* ‘It is hard to think of a process in health care or public health that is not multi-disciplinary and/or cross functional. Referring to multi-disciplinary is a generic feature of improvement (the exceptions are far less frequent than the rule).’
9. Data feedback to implementers	C (6)	• Is continuous feedback an important aspect of the ethical conduct of research? • Is it important to report in publications? • Not feeding back information to those that are part of the QI process may be exploitation by researchers. *Illustrative Comment:* ‘…this seemed self-evident as part of the research, but I struggled to put it in the context of the published paper. I would still rate a 5 to have a description of feedback included because that too, models the process and can act as dissemination of good practice.
10. Specific named improvement methods	A and C (12)	• If an intervention references a specific method it is easier to label as CQI for literature review. • The specific method (e.g. Lean, Six Sigma) varies in how it is used at each site, and projects may combine methods from different models, so the name is not helpful as a defining feature of CQI. *Illustrative Comment: ‘*I think the challenge is how to report methods in a more standardized way.’
11. Set of specific changes	C (10)	• Does ‘set of specific changes’ imply pre-determined changes? If so, not appropriate as CQI. • Is ‘sustaining the gains’ an outcome, or a CQI requirement? *Illustrative Comment:* ‘ … I interpreted this a [stet] merely the importance of embedding improvements into routine work processes. If predetermined were part of the feature then I would definitely rate this extremely low as that would very counterproductive.’

We identified several cross-cutting discussion themes, such as whether it is useful to distinguish between CQI and QI (26 comments). While some felt that distinguishing between QI and CQI is ‘a waste of time,’ because the terms are often used interchangeably in the practice settings, others argued that “QI is not equivalent to CQI…Implementing someone else's intervention may be important, may be laudable, and may represent a significant challenge, but if it is not done iteratively in some manner of PDCA [Plan Do Check Act cycles] then is it CQI? My vote is ‘no’”.

Panelists also posed questions about how the CQI model fits with microlevel process improvement versus macrolevel system improvement in the context of management-initiated changes or concepts of knowledge management (six comments). Three of the six comments indicated that most macrolevel change required microlevel involvement to be successful.

Among 10 comments on the QI theory or framework, two focused on the importance of including ‘customer-mindedness’ or ‘quality in the eyes of the customer.’ Four of the 10 comments focused on publication or reporting and the need for frameworks for this purpose, with the counterbalancing theme of problems with use of labels or jargon.

## Discussion

CQI methods are widely referenced, frequently without many substantiating details. Yet the core meaning of the term remains imprecise. Scientific advancement depends critically on clear specification of methods; when a methodological term has a different meaning from user to user, scientific communication, including comparison with alternative methods, is inefficient. In previous work, we identified 48 different features used to describe CQI in grant proposals and texts; clearly, few CQI efforts will include all 48 [19]. In this study, a large panel of experts from both research and QI practitioner communities agreed that ‘systematic data guided activities,’ ‘designing with local conditions in mind’ and ‘iterative development and testing’ are *essential features of CQI methods*.

The features rated by the experts were phrased to enable abstraction by avoiding use of restrictive terms and promoting the recognition of a wide variety of synonyms that might capture the basic feature. As shown in Table 1, for example, satisfying the criterion ‘systematic data guided activities’ indicated use of something like aims and related measures as the drivers or guides for an initiative's activities. Phrased in these ways, the criteria can identify a core concept that may not have used narrower terms or synonyms. As we previously determined, the presence or absence of features reported on here can be reliably abstracted from QI intervention literature [19].

Based on the work presented here, CQI practitioners, authors, article reviewers and evidence reviews of the literature can be encouraged to reference and report on all three essential CQI features. Panelists also identified seven additional features—‘aiming to change routine work,’ providing ‘data feedback to implementers,’ ‘using evidence relevant to the problem’, having ‘specific predefined aims’ and engaging ‘multidisciplinary teams from target organizations’—as important for CQI success or reporting. Use and reporting of these additional features should also be encouraged when relevant.

Many articles reference specific named methods with CQI and/or industrial TQM roots, such as Lean or Six Sigma [11, 12], often with few additional details about what was done. Based on our quantitative results, use of these terms is of limited usefulness for recognizing CQI. Based on discussion, the terms also have limited methodological meaning to QI experts.

Panelists rated new features from among those panel members proposed during initial discussions. Of these, two were rated as important for reporting on a CQI initiative. These were ‘provides data on what was done to produce change’ and ‘describes the context within which the initiative took place.’ These features focus more on quality of reporting than on recognizing CQI, and have not been tested for reliability. SQUIRE guidelines represent a comprehensive approach to making reporting on QI efforts more systematic [32], and cover substantial material relevant to our panelist-proposed criteria. Our panelists thus indirectly supported two major SQUIRE goals.

A focus of the work reported here is on enabling learning across CQI projects. Given that a single CQI effort for a complex intervention may cost $100 000 per site if carefully audited [33] and that billions of dollars are spent on these efforts nationally, investment in how to harness learning from them would seem to be well spent. Literature review, synthesis and meta-analysis have been a bulwark for learning across healthcare studies. These methodologies require unbiased methods for finding relevant articles. Often, and justifiably, for example, reviews of QI intervention effectiveness, such as those for depression care improvement [34], exclude CQI approaches as being too different from classical intervention literature. Yet studies of CQI, in turn, may not find or include all of the relevant initiatives, given uncertainty about how to recognize them. The three essential CQI features reported here can be used to recognize or screen for use of CQI methods in the literature for evidence syntheses, thus facilitating valid and comprehensive across-study CQI comparisons.

The 11 features rated by our panelists in this study were derived from a previous in-person international expert panel and the literature abstraction process that accompanied it [27]. We showed that, while these features can be reliably abstracted from the literature, there are few articles that include all of them [19]. We also found that articles are so diverse in terms of where they report the features (e.g. abstract, introduction, methods, results or discussion) that full article review was required.

Our previous research shows that retrieving relevant CQI articles from among electronically searched QI intervention articles requires extensive screening [18] and is hampered by the often seemingly random reporting of the CQI activities carried out. These factors limit the completeness, objectivity and coherence with which CQI article sets can be assembled. Stronger consensus among experts and more research on application of QI-related terms may promote better electronic search methods.

This study has limitations. First, an online expert panel approach cannot be assumed to be identical to an in-person panel. The online panel, however, enabled broader validation of the work of our previous in-person international panel; therefore, our study included both panel methods. Moreover, online panelists were generally satisfied with the panel process, found it interesting, easy to use and helpful [25]. Second, while we had some international participants in the ExpertLens process, our sampling aimed at North America. While further work involving the many active CQI researchers and practitioners in other countries is needed, our electronic search strategy yielded many international CQI studies, and the terminology use in these studies is reflected in the panel and reliability work leading up to the online panel. Third, our results may depend in part on how we phrased CQI features, rather than on the features themselves; panelists disagreed with some of our wording. The wording used, however, represents both in-person panel input and several years of work developing a reliable literature abstraction tool [18, 19, 26, 27]. Finally, our participants raised the issue that the terms CQI and QI may be used interchangeably. Our prior work shows the diversity of QI interventions, however, and suggests that distinguishing CQI from the broader term QI will be useful [19, 26].

In summary, we found consensus among a broad group of CQI researchers and practitioners on three features, and three features only, as essential features of CQI methods. Based on these results, we conclude that all three features should be present as a minimum standard for identifying use of CQI methods. The features are (i) using ‘systematic data guided activities’ (e.g. aims and measures) to achieve improvement, (ii) ‘designing with local conditions in mind’ (i.e. to fit the special characteristics of targeted local environment(s)) and (iii) using an ‘iterative development and testing process,’ such as PDSA. These three essential features are validated for use in identifying, designing and reporting on CQI projects.

## Supplementary material

Supplementary material is available at *INTQHC Journal* online.

## Funding

This work was supported by the Robert Wood Johnson Foundation (Grant ID 65113: Advancing the science of continuous quality improvement: a framework for identifying, classifying and evaluating continuous quality improvement studies and Grant ID 67890: Providing a framework for the identification, classification and evaluation of quality improvement initiatives) and the RAND Corporation, with additional funding provided by the VA Health Services Research and Development Center for Implementation Practice and Research Support. Funding to pay the Open Access publication charges for this article was provided by the Department of Veterans Affairs.

## Supplementary Material

Supplementary Data
