# Supplementary material for: How can we recognize continuous quality improvement?
Source: Int J Qual Health Care. 2013 Dec 4;26(1):6–15. doi: 10.1093/intqhc/mzt085 (PMC3914565; doi:10.1093/intqhc/mzt085)
Supplement: Supplementary Data [file supp_26_1_6__index.html]

How can we recognize continuous quality improvement? — Supplementary Data 

# How can we recognize continuous quality improvement?

## Supplementary Data

Supplementary Data

**Files in this Data Supplement:**

- Supplementary Data - Docx file
