# Supplementary material for: How can we recognize continuous quality improvement?
Source: Int J Qual Health Care. 2013 Dec 4;26(1):6–15. doi: 10.1093/intqhc/mzt085 (PMC3914565; doi:10.1093/intqhc/mzt085)
Supplement: Supplementary Data [file supp_mzt085_mzt085supp.docx]

Online Material

**Appendix Table 1:** Feature importance to definition, CQI success and reporting

| **Feature** | **Importance to the Definition** | | **Importance to CQI Success** | | **Importance of Reporting the Specifics in Publication** | |
| --- | --- | --- | --- | --- | --- | --- |
|  | **Number of Panels Reaching Consensus (out of four panels total)** | **% Rated Highly Important Across All Panelists** | **Number of Panels Reaching Consensus (out of four panels total)** | **% Rated Highly Important Across All Panelists** | **Number of Panels Reaching Consensus (out of four panels total)** | **% Rated Highly Important Across All Panelists** |
| **1 Systematic Data Guided Activities** | **4** | **99%** | **4** | **96%** | **4** | **98%** |
| 2 Aiming to Change Routine Work | 3 | 76% | 4 | 81% | 4 | 76% |
| 3 Creating a Culture of Quality Improvement | 0 | 52% | 3 | 81% | 0 | 37% |
| 4 Specific, Pre-Defined Aims | 3 | 83% | 4 | 84% | 3 | 88% |
| 5. Using Evidence Relevant to the Problem | 2 | 63% | 1 | 62% | 4 | 71% |
| **6. Designing with Local Conditions in Mind** | **4** | **90%** | **4** | **95%** | **4** | **85%** |
| **7. Iterative Development and Testing** | **4** | **85%** | **4** | **88%** | **4** | **80%** |
| 8. Multidisciplinary Teams from Target Organizations | 3 | 66% | 4 | 80% | 3 | 72% |
| 9. Data Feedback to Implementers | 3 | 84% | 4 | 99% | 4 | 83% |
| 10. Specific Named Improvement Methods | 0 | 37% | 0 | 38% | 2 | 61% |
| 11. Set of Specific Changes | 3 | 77% | 3 | 82% | 3 | 82% |

Note: All four panels independently and all participants taken together consider the bold features important to the definition, success, and reporting.
